# ANP32 Proteins Are Essential for Influenza Virus Replication in Human Cells

**DOI:** 10.1128/JVI.00217-19

**Published:** 2019-08-13

**Authors:** Ecco Staller, Carol M. Sheppard, Peter J. Neasham, Bhakti Mistry, Thomas P. Peacock, Daniel H. Goldhill, Jason S. Long, Wendy S. Barclay

**Affiliations:** a Section of Molecular Virology, Imperial College London, St Mary’s Campus, London, United Kingdom; Icahn School of Medicine at Mount Sinai

**Keywords:** ANP32, ANP32A, ANP32B, CRISPR, host factor, influenza, polymerase, replication, virus

## Abstract

ANP32 proteins have been implicated in supporting influenza virus replication, but most of the work to date has focused on the ability of avian Anp32 proteins to overcome restriction of avian influenza polymerases in human cells. Using a CRISPR approach, we show that the human acidic nuclear phosphoproteins (ANPs) ANP32A and ANP32B are functionally redundant but essential host factors for mammalian-adapted influenza A virus (IAV) and influenza B virus (IBV) replication in human cells. When both proteins are absent from human cells, influenza polymerases are unable to replicate the viral genome, and infectious virus cannot propagate. Provision of exogenous ANP32A or ANP32B recovers polymerase activity and virus growth. We demonstrate that this redundancy is absent in the murine Anp32 orthologues; murine Anp32A is incapable of recovering IAV polymerase activity, while murine Anp32B can do so. Intriguingly, IBV polymerase is able to use murine Anp32A. We show, using a domain swap and point mutations, that the leucine-rich repeat (LRR) 5 region comprises an important functional domain for mammalian ANP32 proteins. Our approach has identified a pair of essential host factors for influenza virus replication and can be harnessed to inform future interventions.

**IMPORTANCE** Influenza virus is the etiological agent behind some of the most devastating infectious disease pandemics to date, and influenza outbreaks still pose a major threat to public health. Influenza virus polymerase, the molecule that copies the viral RNA genome, hijacks cellular proteins to support its replication. Current anti-influenza drugs are aimed against viral proteins, including the polymerase, but RNA viruses like influenza tend to become resistant to such drugs very rapidly. An alternative strategy is to design therapeutics that target the host proteins that are necessary for virus propagation. Here, we show that the human proteins ANP32A and ANP32B are essential for influenza A and B virus replication, such that in their absence cells become impervious to the virus. We map the proviral activity of ANP32 proteins to one region in particular, which could inform future intervention.

## Abstract (precis)

Influenza virus is the etiological agent behind some of the most devastating infectious disease pandemics to date, and influenza outbreaks still pose a major threat to public health. Influenza virus polymerase, the molecule that copies the viral RNA genome, hijacks cellular proteins to support its replication. Current anti-influenza drugs are aimed against viral proteins, including the polymerase, but RNA viruses like influenza tend to become resistant to such drugs very rapidly. An alternative strategy is to design therapeutics that target the host proteins that are necessary for virus propagation. Here, we show that the human proteins ANP32A and ANP32B are essential for influenza A and B virus replication, such that in their absence cells become impervious to the virus. We map the proviral activity of ANP32 proteins to one region in particular, which could inform future intervention.

## INTRODUCTION

Influenza viruses are a major cause of respiratory illness and mortality worldwide, causing approximately 500,000 deaths annually from seasonal epidemics alone (1). An additional and potentially much more serious burden arises from zoonotic emergence of pandemic viruses from birds, the natural reservoir of influenza viruses. The most notorious of these events, in 1918, claimed the lives of an estimated 50 million people, while the latest pandemic to date, in 2009, killed over 250,000 worldwide (2). In order to mitigate the impact of the next influenza pandemic and reduce the seasonal burden, new approaches to thwart influenza virus are required. A first step toward novel treatment is an enhanced understanding of the interactions between virus and host cell.

Influenza virus requires the host cell machinery to support replication of its genome and production of new virions. The influenza genome is made up of eight segments of single-stranded negative-sense RNA (vRNA). Each segment is packaged in a double helical loop structure bound by nucleoprotein (NP) along its length, except for the pseudocomplementary 3′ and 5′ untranslated regions that comprise the promoter (3–5). These termini instead associate with an RNA-dependent RNA polymerase (RdRp) encoded by the virus (6). This key enzyme, a heterotrimer of polymerase basic protein 1 (PB1), polymerase basic protein 2 (PB2), and polymerase acidic protein (PA), functions as both a transcriptase and a replicase (reviewed by te Velthuis and Fodor [7]). Transcription of mRNA and replication through a positive-sense cRNA take place in the host cell nucleus (7, 8). A viral complex containing RNA, NP, and RdRp is termed a ribonucleoprotein (RNP), which, depending on the sense of the RNA, is either a vRNP or a cRNP.

ANP32A and ANP32B are small acidic nuclear phosphoproteins (ANPs) that are attributed to a plethora of cellular functions (9), including chromatin remodeling (10, 11), apoptosis (12, 13), transcription regulation (14, 15), and intracellular transport (16). ANP32 proteins are approximately 250 amino acids in length and contain an N-terminal leucine-rich repeat (LRR) region, a central domain, and an unstructured low-complexity acidic region (LCAR) at the C terminus. ANP32 proteins have been associated with influenza A polymerase function. A nuclear fraction containing ANP32A and ANP32B was shown to enhance the synthesis *in vitro* of vRNA from a short cRNA template (17). Knockdown of ANP32A or ANP32B in human cells reduced polymerase activity measured in minigenome reporter assays, as well as synthesis of viral RNA in infected cells (17, 18). Direct interactions of ANP32 proteins with the RdRp or RNP have been documented but do not completely correlate with function (19–22). The difference between avian and mammalian ANP32A proteins has been suggested to account for host range restriction of avian influenza strains in mammalian cells, and much of the work to date has focused on the avian orthologues, particularly those from chickens (23).

Here, we use CRISPR/Cas9 genome editing to render the *Anp32A* and/or *Anp32B* genes nonfunctional in low-ploidy human eHAP1 cells (24, 25), thus obtaining a clean experimental platform in which to investigate the interplay between different influenza virus polymerases and mammalian ANP32 proteins. We find that although IAV and IBV polymerases can replicate in the absence of either ANP32A or ANP32B alone (i.e., in single-knockout cells), depletion of both proteins (double knockout) renders the cell impervious to RdRp activity. Furthermore, none of the IAV strains tested is capable of replication in the double knockout cells. Human ANP32A and ANP32B proteins are thus functionally redundant but essential for influenza virus replication. We further show that this redundancy is not present in the murine Anp32 orthologues. Only murine Anp32B (MusB) is able to recover IAV polymerase activity, although, surprisingly, murine Anp32A (MusA) can be coopted by IBV polymerase. Functionality mapped to leucine-rich repeat 5 of the LRR domain, thus assigning this domain of the host proteins as key for the support of influenza polymerase activity and a target for future interventions.

## RESULTS

### Generation of eHAP1 knockout cells.

**FIG 1:**
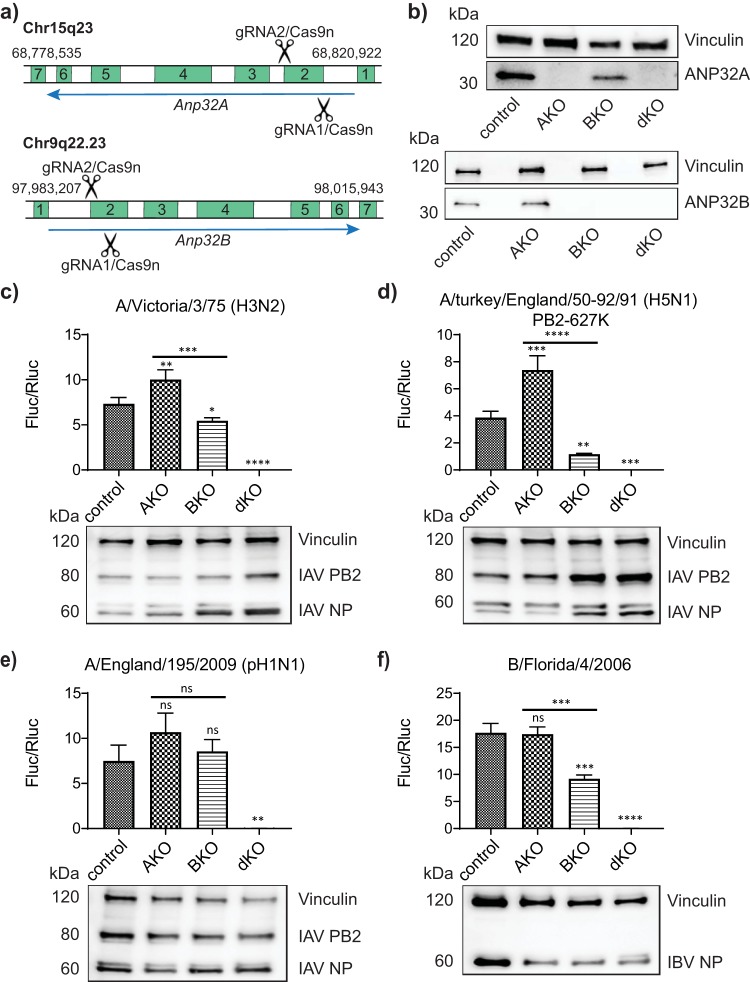
Human cells lacking ANP32A and ANP32B do not support influenza virus polymerase activity. (a) Schematic showing location of CRISPR guide RNA target sequences in the gene structure of human ANP32A or ANP32B. (b) Western blotting showing ANP32A (upper panels) or ANP32B (lower panels) expression in eHAP1 control, single- (AKO and BKO) and double- (dKO) knockout cells. (c to f) Minigenome assays in eHAP1 control, AKO, BKO, or dKO cells. Cells were transfected with plasmids to reconstitute polymerase from H3N2 Victoria (c), H5N1 50-92 (PB2 627K) (d), pH1N1 England 195 (e), or IBV Florida 06 (f) virus, along with IAV or IBV firefly minigenome reporter and *Renilla* expression control. Data shown are firefly activity normalized to *Renilla*, plotted as mean (standard deviation [SD]) obtained by one-way analysis of variance (ANOVA) from one representative repeat (*n* > 3). Accompanying Western blots show expression of respective vRNP components in each cell type (representative of one minigenome assay). ns, not significant; *, *P* < 0.05; **, *P* < 0.01; ***, *P* < 0.001; ****, *P* < 0.0001.

eHAP1 cells lacking ANP32A (AKO), ANP32B (BKO), or both proteins (dKO) were generated by CRISPR/Cas9 genome editing using a double-nickase approach for enhanced specificity and minimal off-target DNA cleavage (26, 27) (Fig. 1a). Control cells (control) were treated in an identical manner with nontargeting guide RNAs (28). Two independent clones with diallelic disruption of the *Anp32A* or *Anp32B* locus were verified by next-generation sequencing (NGS) and Sanger sequencing of individual alleles, and loss of protein expression was confirmed by Western blotting against ANP32A or ANP32B, respectively (Fig. 1b and data not shown). Double-knockout cells were generated by tandem CRISPR from a BKO clone, using the guides against the *Anp32A* locus. Three independent dKO clones were verified by Sanger sequencing, and loss of expression was confirmed by Western blot analysis (Fig. 1b and data not shown).

### Influenza virus polymerase activity is dependent on either ANP32A or ANP32B.

Minigenome reporter assays were carried out in single and double ANP32-knockout cells with reconstituted polymerases from three different influenza A viruses—a seasonal H3N2 virus (A/Victoria/3/75), an avian H5N1 virus (A/turkey/England/50-92/1991) with the mammalian-adapting mutation E627K in the PB2 subunit, and a 2009 pandemic H1N1 virus (A/England/195/2009)—as well as an influenza B virus (B/Florida/04/2006). Surprisingly, absence of ANP32A in human cells did not result in loss of influenza polymerase activity; in fact, activity increased for some polymerase constellations. (Fig. 1c to f). Polymerase activity in BKO clones was either unaffected or decreased but not abrogated (Fig. 1c to f). A similar pattern was observed in A549 cells lacking ANP32A or ANP32B (data not shown). Strikingly, however, none of the polymerases showed any activity in three independent double-knockout lines (Fig. 1c to f and data not shown), despite robust expression of vRNP components (Fig. 1c to f). These data suggest functionally redundant roles for ANP32A and ANP32B in supporting influenza virus polymerase activity in human cells.

**FIG 2:**
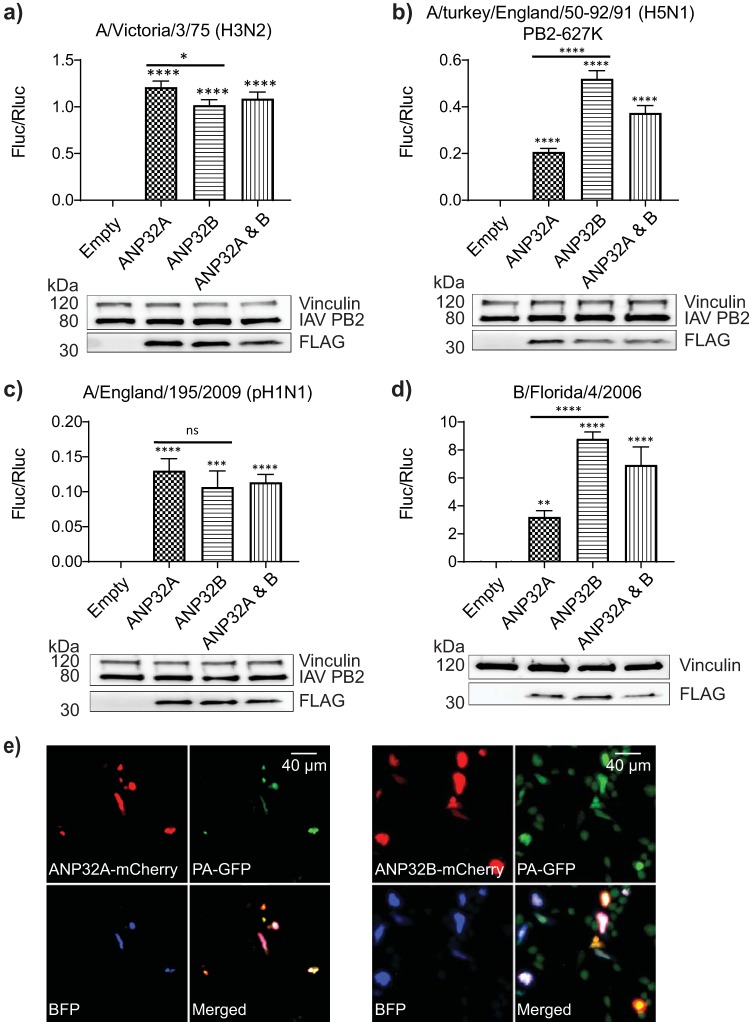
Exogenous ANP32 expression recovers polymerase activity in dKO cells. (a to d) Minigenome assays in eHAP1 dKO cells with coexpressed Empty vector (Empty), FLAG-tagged ANP32A, ANP32B, or ANP32A and ANP32B expression plasmids. Accompanying Western blots show expression of FLAG-tagged ANP32 constructs alongside respective vRNP components (representative of one minigenome assay). Data shown are firefly activity normalized to *Renilla* plotted as mean (SD) obtained by one-way ANOVA from one representative repeat (*n* > 3). Ns, not significant; **, *P* < 0.01; ****, *P* < 0.0001. (e) Expression from IAV minigenome encoding NLS-tagged BFP in eHAP1 dKO cells exogenously reconstituted with mCherry-tagged ANP32A or ANP32B. Cells were transfected with expression plasmids encoding 50-92 PB1, PB2 627K, and PA-GFP, as well as NP, and either mCherry-tagged ANP32A or ANP32B and a blue fluorescent protein (BFP-NLS) minigenome reporter.

In order to confirm redundancy, polymerases were coexpressed in dKO cells with plasmids encoding exogenous ANP32A, ANP32B, or equal amounts of both. All polymerases tested regained activity in the presence of either ANP32 protein (Fig. 2a to d). Provision of both proteins at once did not further enhance rescue. These results were corroborated at the single-cell level: ANP32A or ANP32B proteins fused to the red fluorescent protein mCherry were coexpressed in dKO cells with H5N1 (PB2 627K) 50-92 vRNP components PB1, PB2-627K, PA-green fluorescent protein (GFP), and NP, with an influenza minigenome encoding blue fluorescent protein (BFP) as a reporter. Blue fluorescence resulting from active polymerase was observed only in cells that expressed ANP32 proteins, and either paralogue was able to rescue activity (Fig. 2e).

These data demonstrate that ANP32A and ANP32B proteins are essential but redundant for influenza A and B polymerase activity in human cells. Intriguingly, we observed that recovery of polymerase activity in dKO cells was more efficiently achieved by expression of ANP32B than ANP32A for specific polymerase constellations, namely avian H5N1 50-92 (PB2 627K) and IBV Florida 06 (Fig. 2b and d). These two polymerase constellations were also more affected by loss of ANP32B expression (Fig. 1d and f), suggesting ANP32B is the preferred host factor for these polymerases in human cells.

### IAV replication is abrogated in cells lacking ANP32A and ANP32B.

**FIG 3:**
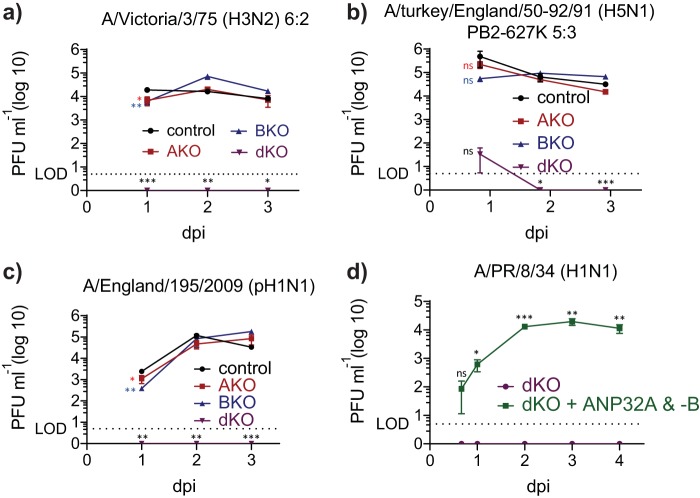
IAV replication is abrogated in dKO cells. (a to c) Control (black), AKO (red), BKO (blue), and dKO (purple) cells were infected with H3N2 Victoria 6:2 reassortant virus with PR8 hemagglutinin (HA) and NA genes, H5N1 A/Tky/50-92 (PB2-627K) 5:3 reassortant virus with PR8 HA, neuraminidase (NA), and matrix protein (M) genes, or pH1N1 England 195, respectively (MOI = 0.005) and incubated at 37°C in the presence of 1 μg/ml trypsin to allow multicycle replication. Supernatants were harvested at the indicated number of days postinfection (dpi) and PFU · ml^−1^, established by plaque assay on MDCK cells. (d) dKO cells were transfected with equimolar amount of ANP32A and ANP32B expression plasmids 6 h prior to infection with H1N1 PR8 virus (MOI = 0.005). Cells were incubated at 37°C in the presence of 1 μg/ml trypsin, and supernatants were collected at indicated time points. Data shown are mean PFU · ml^−1^ measured by plaque assay on MDCK cells. LOD (dotted line) denotes the limit of detection based on the dilution factor in plaque assays. All infection experiments were repeated at least twice. Graphs shown are of one representative triplicate assay. Statistical significance was calculated per time point by Student’s *t* test. Ns, not significant; *, *P* < 0.05; **, *P* < 0.01; ***, *P* < 0.001; ****, *P* < 0.0001.

To investigate the consequence of absence of ANP32A or ANP32B proteins on infectious IAV replication in human cells, control, single-knockout, and double-knockout cells were infected at a multiplicity of infection (MOI) of 0.005 with three different viruses whose genetic content corresponded to the polymerase constellations tested in Fig. 1 and 2, i.e., from Vic/75, Tky/50-92 (E627K), or Eng195. While virus replicated to high titers in control and single-knockout (KO) cells, replication in dKO cells was completely abrogated (Fig. 3a to c). This suggests that viral proteins such as NEP and NS1 (expressed during viral infection but not provided in the minigenome assay) cannot overcome the block in replication imposed by the absence of ANP32 proteins. Replication of the H1N1 laboratory-adapted strain A/PR/8/34 was also abrogated in dKO cells (data not shown). Reconstitution of dKO cells with both ANP32A and ANP32B proteins by transient transfection prior to infection restored PR8 virus replication (Fig. 3d).

**FIG 4:**
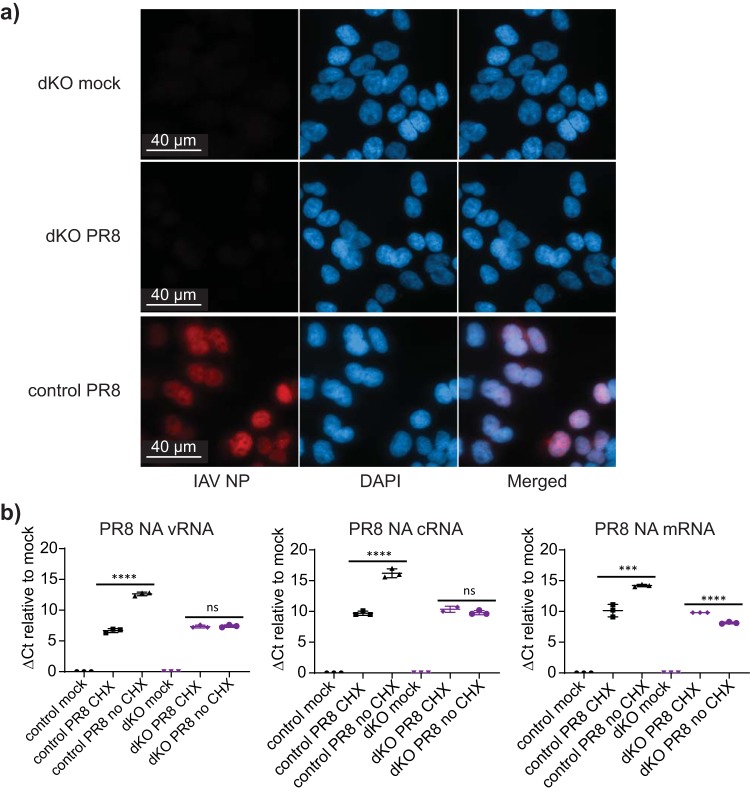
Synthesis of vRNA from the cRNA template is abrogated in cells lacking ANP32A and ANP32B. **(**a) Immunofluorescence analysis for NP expression in control cells, dKO, or mock-infected cells. Cells were infected with H1N1 PR8 virus (MOI = 0.2) for 5 h in growth medium, then fixed and incubated with primary α-IAV NP antibody followed by Alexa Fluor-568 secondary antibody and imaged on a Zeiss Cell Observer widefield microscope. The lower detection limit in the TexasRed channel was set to that of the DAPI channel. (b) qRT-PCR analysis demonstrating accumulation of PR8 virus segment 6 vRNA, cRNA, and mRNA in the absence or presence of 100 μg · ml^−1^ cycloheximide (CHX). eHAP1 control or dKO cells were transfected with H5N1 Tky/50-92 polymerase components PB1-D446Y (catalytically inactive), PB2-627K, and PA in a 1:1:1 ratio 20 h prior to infection with PR8 virus at an MOI of 10. RNA was extracted 5 h postinfection. Data show mean (SD) of 40-*C_T_* values normalized to mean mock-infected levels, analyzed per cell type by one-way ANOVA. Experimental data are representative of 3 repeats. ns, not significant; ***, *P* < 0.001; ****, *P* < 0.0001.

It has been suggested that ANP32A and ANP32B specifically support the synthesis of negative-sense vRNA from a positive-sense intermediate template (cRNA) (17). This is believed to occur after primary transcription in *cis* of the vRNA by the incumbent RdRp and requires newly synthesized RdRp molecules to stabilize the cRNA in *trans* (29, 30). Therefore, without replication, secondary transcription and accumulation of viral proteins will not occur. We used immunofluorescence microscopy to visualize accumulation of viral nucleoprotein (NP) in control and dKO cells 5 h postinfection with H1N1 PR8 virus. NP accumulation exceeded background level only in cells containing ANP32 proteins (Fig. 4a), but this approach was not sufficiently sensitive to image NP protein products of primary transcription. In order to assess which viral RNAs were synthesized in cells that lack expression of ANP32 proteins, we preexpressed an inactive influenza polymerase complex (to stabilize any cRNA generated) for 20 h before infecting with high-MOI virus in presence or absence of cycloheximide (CHX). Five hours later, levels of vRNA, cRNA, and mRNA generated from segment 6 of the incoming virus were assayed by reverse transcription-quantitative PCR (qRT-PCR) (Fig. 4b). In control cells, amplification of all 3 RNA species was evident in the absence of CHX. In dKO cells, RNA levels were not different in presence or absence of CHX, indicating that vRNA was not replicated. Primary transcription of mRNA and pioneering round generation of cRNA (i.e., replication of cRNA from the incoming vRNA by the incumbent *cis* RdRp) were detected in dKO cells, since RNA levels were similar to those detected in the presence of CHX in control cells. Thus, our data support the block to replication occurring at the step of copying cRNA back to vRNA in the absence of ANP32 proteins.

### Murine Anp32B supports IAV polymerase.

**FIG 5:**
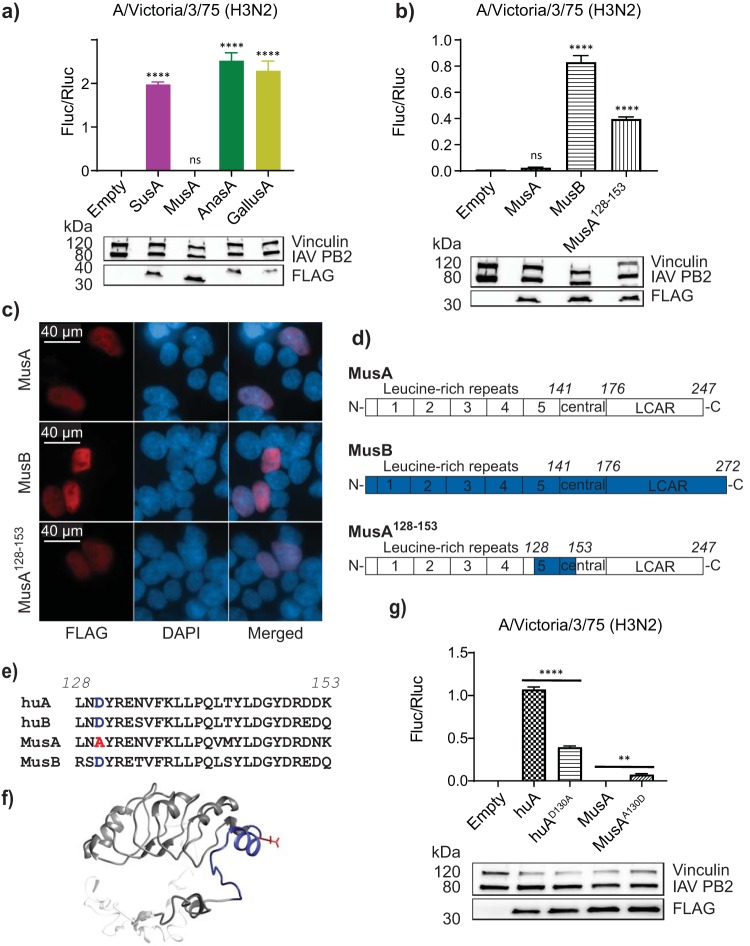
Influenza A virus polymerase activity is supported by murine Anp32B but not by Anp32A. (a) Minigenome reporter assay in dKO cells with cotransfected FLAG-tagged Anp32A from pig (SusA), mouse (MusA), duck (AnasA), or chicken (GallusA) with H3N2 Victoria RNP components, pPolI-firefly minigenome reporter, and *Renilla* transcription control. Data show mean (SD) of firefly activity normalized to that of *Renilla* and analyzed by one-way ANOVA from one representative repeat (*n* = 2 triplicate experiments). ns, not significant; ****, *P* < 0.0001. Accompanying Western blot shows expression of vRNP component PB2 and coexpressed FLAG-tagged ANP32 constructs. (b) Minigenome assay showing activity of Vic/75 polymerase in eHPA1 dKO cells coexpressing mouse Anp32A, Anp32B, or Anp32A^128-153^ Data were analyzed as described for panel a. Accompanying Western blot shows expression of vRNP component PB2 and coexpressed FLAG-tagged ANP32 constructs. (c) Immunofluorescence analysis showing nuclear expression of FLAG-tagged MusA, MusB, and MusA^128-153^ detected with anti-FLAG antibody and Alexa Fluor-594 anti-rabbit conjugate and counterstained with DAPI. (d) Cartoon showing chimeric mouse ANP32 protein with 26 amino acids from mouse Anp32A LRR 5 replaced by the equivalent sequence from mouse Anp32B. (e) Alignment comparing LRR 5 amino acid sequence of mouse Anp32A to its Anp32B homologue, human ANP32A, and human ANP32B. (f) Structural model of mouse Anp32B highlighting the swapped domain in blue (LRR in dark gray, LCAR in semitransparent gray, and the domain swap in blue with amino acid 130 represented as a red stick). (g) Minigenome assay for activity of H3N2 Victoria polymerase in eHAP1 dKO cells coexpressing wild-type human or mouse ANP32A or position 130 point mutants. Data show mean (SD) of firefly activity normalized to that of *Renilla* and analyzed by one-way ANOVA from one representative repeat (*n* = 3 triplicate experiments). **, *P* < 0.01; ****, *P* < 0.0001. Accompanying Western blot shows expression of vRNP component PB2 and coexpressed FLAG-tagged ANP32 constructs.

We used the complementation assay in dKO cells to ask whether Anp32 proteins from nonhuman species relevant to influenza virology were capable of supporting polymerase function. We carried out minigenome reporter assays coexpressing Anp32A proteins from pig (SusA), mouse (MusA), duck (AnasA), and chicken (GallusA) with IAV polymerase (H3N2 Victoria/75). While the avian and porcine orthologues could support IAV polymerase, MusA could not (Fig. 5a). Bearing in mind that our results suggest that human ANP32B might be the more potent host factor for some polymerase constellations, we hypothesized that in mice, influenza virus might rely solely on MusB to support its replication. Indeed, expression of MusB could recover Vic/75 polymerase activity in dKO cells (Fig. 5b), despite equal levels of expression of both murine Anp32 proteins and their localization to the cell nucleus (Fig. 5b and c).

An alignment of murine and human ANP32 proteins showed several unique features in the MusA sequence, mapping largely to surface-exposed residues within LRR 5 (Fig. 5d). In order to determine whether these differences were responsible for the lack of functionality of MusA, we generated a chimera of murine Anp32A and B (MusA^128-153^) by substituting a 26-amino acid (aa) segment (aa 128 to 153) of MusB into MusA (Fig. 5d), and then tested if this conferred gain of function on MusA to support IAV polymerase. The chimera was indeed capable of recovering activity of Vic/75 polymerase in dKO cells, although not to the level shown by MusB (Fig. 5b).

Western blot and immunofluorescence analysis of the FLAG-tagged chimeric construct demonstrated expression and nuclear localization (Fig. 5 b and c).

We identified a single amino acid in LRR 5 at position 130 that was the same in human ANP32A or ANP32B and MusB (aspartic acid, D) but which differed in MusA (alanine, A) (Fig. 5e and f). Introduction of a D130A single-point mutation in human ANP32A significantly reduced its ability to support Vic75 polymerase activity, and conversely, introduction of A130D to MusA produced a small but significant increase in its ability to support viral polymerase (Fig. 5g).

**FIG 6:**
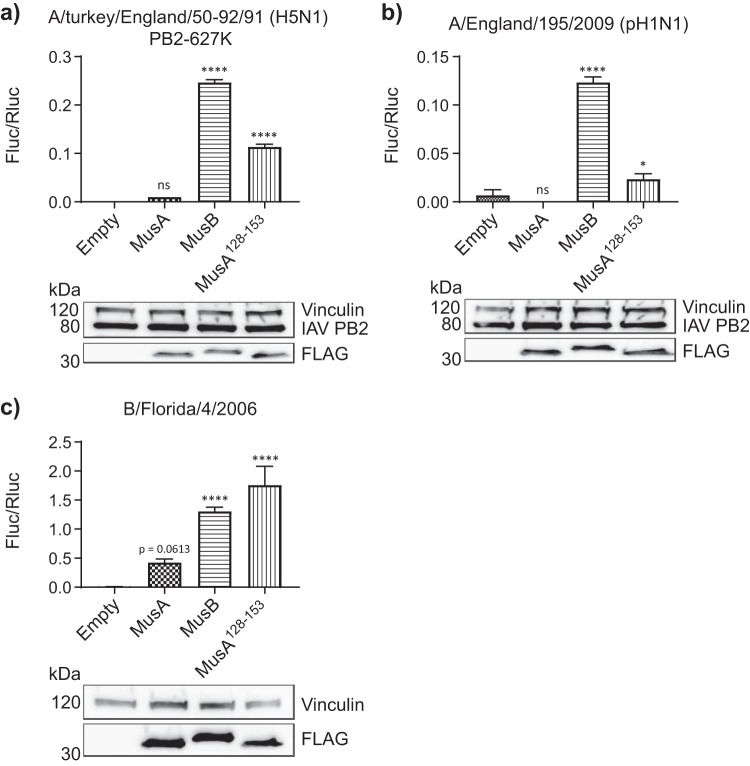
Murine Anp32A can support IBV but not IAV polymerase activity. Minigenome assays in eHAP1 dKO cells showing activity of polymerases from H5N1 Tky/50-92 (PB2-627K) (a), pH1N1 Eng/195 (b), and B/Florida/06 (c) cotransfected with FLAG-tagged mouse Anp32A, Anp32B, or Anp32A^128-153^. Data show mean (SD) of firefly activity normalized to *Renilla* and analyzed by one-way ANOVA from one representative repeat (*n* = 2 triplicate experiments). ns, not significant; *, *P* < 0.05; ****, *P* < 0.0001. Accompanying Western blot shows expression of vRNP component PB2 and coexpressed FLAG-tagged ANP32 constructs.

Finally, we explored whether MusA or MusB could support activity of polymerases derived from other IAV strains or from IBV. As seen for Vic/75 polymerase, MusA was nonfunctional for IAV polymerases from A/Tky/50-92/91 and A/Eng/195 (Fig. 6a and b); however, IBV Florida 06 polymerase recovered some activity in dKO cells in the presence of MusA (Fig. 6c). MusB, however, was the more potent factor for support of IBV polymerase. Intriguingly, IBV Florida 06 polymerase activity was even greater in the presence of the MusA/MusB chimera than in that of MusB alone (Fig. 6c).

## DISCUSSION

Here, we show that human ANP32A and ANP32B are functionally redundant in their support for influenza virus polymerase in human cells and that the RdRp does not carry out RNA replication in the absence of both family members. Our findings corroborate those of Zhang et al. (2019) who used a similar CRISPR approach (31) We further show that IBV polymerase is also dependent on human ANP32 proteins and can also utilize either orthologue to support activity. Our demonstration of redundancy in use of these essential host factors illuminates a deficiency in RNA interference (RNAi) or CRISPR screens where host genes are knocked down one at a time. Functionally redundant pairs or larger groups of host factors that can be used by a virus will escape detection, as have ANP32A and ANP32B individually in previous screens (32, 33).

Two observations imply preference of certain polymerase constellations for human ANP32B over ANP32A. First, specific polymerases were more efficiently enhanced by ANP32B when provided exogenously. Second, the absence of ANP32A in the cell enhances virus polymerase activity in some cases, where the opposite might be expected and has previously been observed in knockdown experiments (17, 18). The latter observation might be explained if ANP32B is held in heterodimers or larger protein complexes with ANP32A, thus absence of ANP32A might liberate the preferred ANP32B for recruitment by influenza virus RdRp. Alternatively, ANP32A and ANP32B, being very similar structurally, might compete for polymerase binding, and loss of ANP32A would then favor binding and more efficient activity mediated by ANP32B. Taken together, the observations point to ANP32B being functionally superior in humans to ANP32A for supporting influenza polymerase. However, these differences were not so readily apparent in the context of virus infection where replication continued largely unabated in single ANP32A or ANP32B knockout cells.

Interestingly, the redundancy observed in humans is not observed for murine Anp32 proteins. IAV polymerases cannot use MusA, but IBV polymerase can, albeit inefficiently. The ability of influenza A virus to replicate in mice is explained by the utility of murine Anp32B. Mapping this difference using a chimeric approach revealed LRR 5 as a key domain of ANP32 proteins for supporting IAV polymerase, and point mutation highlighted the role of a single amino acid in LRR 5 at position 130. It will be important to investigate whether this is a contact point in the interaction between the viral complex and the host protein, and this will be a crucial question for structural studies to address. The highlighted domain sits adjacent to a linker region between the structured LRR and highly flexible LCAR, and it may be important for defining overall structural arrangement and susceptibility to conformational change. It is interesting to note that in chicken cells, it is the Anp32A orthologue that is utilized by avian influenza polymerase, whereas chAnp32B does not support replication; this difference in functionality was also mapped to LRR 5 (31, 34).

Current anti-influenza therapeutics, as well as drugs in development such as adamantanes (M2 ion channel inhibitors), neuraminidase inhibitors (NAIs) such as oseltamivir, and RdRp-targeting molecules (including the nucleoside analogue favipiravir and small molecules such as baloxavir), are all aimed at proteins encoded by the virus. A recurring issue with such drugs is the ease with which influenza virus evolves resistance to them, be it in a laboratory setting (35, 45) or in the field (36–38). An alternative approach would be to target specific interactions of virus proteins with essential host factors, such that small-molecule inhibitors may temporarily block the interacting surface on the host protein without compromising its cellular functions. As influenza virus replication is completely abrogated in their absence, ANP32 proteins suggest themselves as potential candidates for such an approach.

## MATERIALS AND METHODS

### Cells and cell culture.

Human eHAP1 cells (Horizon Discovery) were cultured in Iscove’s modified Dulbecco’s medium (IMDM; Thermo Fisher) supplemented with 10% fetal bovine serum (FBS; Labtech), 1% nonessential amino acids (NEAA; Gibco), and 1% penicillin/streptomycin (Invitrogen). Human lung adenocarcinoma epithelial cells (A549) (ATCC) and Madin-Darby canine kidney (MDCK) cells (ATCC) were maintained in Dulbecco’s modified Eagle’s medium (DMEM; Invitrogen) supplemented with 10% FBS, 1% NEAAs, and 1% penicillin-streptomycin (Invitrogen). All cells were maintained at 37°C in a 5% CO_2_ atmosphere.

### Plasmids and cloning.

cDNAs of full-length human codon-optimized murine *Anp32A* and *Anp32B* isoforms were generated by gene synthesis (GeneArt, Thermo Fisher) using GenBank sequences NP_033802.2 (mouse *Anp32A*) and NP_570959.1 (mouse *Anp32B*) and cloned into pCAGGS expression plasmids that included a C-terminal GSG linker followed by a FLAG tag and 2 stop codons. Human pCAGGS *ANP32A* and *ANP32B*, chicken *Anp32A*, pig *Anp32A*, and duck *Anp32A* expression plasmids have been previously described (18). The chimeric mouse construct and human ANP32A point mutants were cloned by overlapping PCR, using primers CCAACCTGAATGCCTACCGCGAGAAC and GTTCTCGCGGTAGGCATTCAGGTTGG (huANP32A-D130A), and GGTCACTTCGCAGTTAAACAAATCCAG, GTTTAACTGCGAAGTGACCAACAGAAGC, GCCCTCCACGTCGCTGTCAGGGGCCTC, and GACAGCGACGTGGAGGGCTACGTGGAG (mouse Anp32A/Anp32B domain swap). Mouse ANP32A mutant A130D was generated by overlapping PCR using primers GGTAACCAACCTTAATGATTACCGGGAGAACGTC and GACGTTCTCCCGGTAATCATTAAGGTTGGTTACC. pCAGGS expression plasmids encoding each polymerase component and NP for H3N2 Victoria, H5N1 (PB2-627K) 50-92, pH1N1 England 195, and IBV Florida 06 have been described (39, 40) pPolI reporter plasmids containing firefly luciferase or blue fluorescent protein flanked by IAV- or IBV-specific promoters have been previously described (41). All plasmid constructs were verified by Sanger sequencing and analyzed manually in Geneious v6.

### Generation and screening of CRISPR clones.

Pairs of guide RNAs against exon 2 of human *Anp32A* (GTCAGGTGAAAGAACTTGTCC and GAAGGCCCGACCGTGTGAGCG) and *Anp32B* (GAGCCTACATTTATTAAACTG and GCAAGCTGCCTAAATTGAAAA) were designed with the aid of the CRISPR design tool at www.crispr.mit.edu (Feng Zhang Lab). The nontargeting guide RNA pair was GTATTACTGATATTGGTGGG and GAACTCAACCAGAGGGCCAA. The guides were cloned into plasmid pSpCas9n(BB)-2A-Puro (PX462) v2.0 (Feng Zhang Lab), obtained via Addgene, and equimolar amounts of plasmids were transfected using Lipofectamine 3000 (Thermo Fisher). Cells harboring at least one plasmid were enriched by selection with puromycin at 1.5 μg · ml^−1^ for 3 to 5 days and single-cell sorted into 96-well plates containing growth medium, using a fluorescence-activated cell sorter (FACS) Aria IIIU (BD Biosciences) with an 85-μm nozzle. Single cells were grown out into clonal populations over a period of 10 to 14 days. Genetic loci harboring insertion/deletion mutations (indels) were amplified by PCR using barcoded primers (AGTGACGGAGTGACTGACTG and GAGGTGAGGCCTACGTTGAT for *Anp32A*; TGTCTTGGACAATTGCAAATCAA and CCATGTGCTTTCTGCTACACT for *Anp32B*) (42). A total of 268 barcoded amplicons were then prepared for next-generation sequencing (NGS) using the NEBNext Ultra II kit (NEB) and sequenced using 150-bp paired-end reads on an Illumina MiSeq instrument. Reads were mapped using Burrows-Wheeler Aligner (BWA) v0.7.5. Indels occurring above a cutoff of 2.5% of reads were detected using an R script (https://github.com/Flu1/CRISPR). DNA sequences were analyzed in Geneious v6.

### Immunoblot analysis.

At least 250,000 cells were lysed in buffer containing 50 mM Tris-HCl (pH 7.8; Sigma-Aldrich), 100 mM NaCl, 50 mM KCl, and 0.5% Triton X-100 (Sigma-Aldrich), supplemented with a cOmplete EDTA-free protease inhibitor cocktail tablet (Roche) and prepared in Laemmli 2× buffer (Sigma-Aldrich) after protein concentration had been established by spectrophotometry (NanoDrop; Thermo Fisher). Equal amounts of total protein were resolved by SDS-PAGE using Mini Protean TGX precast gels 4% to 20% (Bio-Rad). Immunoblotting by semidry transfer (Bio-Rad Trans-Blot SD semidry transfer cell) onto nitrocellulose membranes (Amersham Protran Premium 0.2-μm NC; GE Healthcare) was carried out using the following primary antibodies: rabbit α-vinculin (catalog number ab129002, 1/1,000; Abcam), rabbit α-ANP32A (catalog number ab51013, 1/500; Abcam), mouse α-ANP32B (66160-1-Ig, 1/1,000; Proteintech) or rabbit α-ANP32B (10843-1-AP, 1/1,000; Proteintech), mouse α-IAV NP (catalog number ab128193, 1/1,000; Abcam), mouse α-IBV NP (catalog number ab20711, 1/1,000; Abcam), mouse α-FLAG (catalog number F1804, 1/500; Sigma-Aldrich), and rabbit α-IAV PB2 (catalog number GTX125926, 1/4,000; GeneTex). The following secondary antibodies were used: sheep α-rabbit horseradish peroxidase (HRP) (catalog numberAP510P, 1/10,000; Merck) and goat α-mouse HRP (STAR117P, 1/5,000; AbD Serotec). Protein bands were visualized by chemiluminescence (ECL Prime Western blotting detection reagent; GE Healthcare) using a Fusion-FX imaging system (Vilber Lourmat). Western blots in Fig. 1c to f, 2a to d, 5a, 6a to d, and 7c represent the accompanying minigenome assay; Western blots in Fig. 1b are representative of at least 3 repeats.

### Minigenome assay.

In order to measure influenza virus polymerase activity, pCAGGS expression plasmids encoding PB1 (0.04 μg), PB2 (0.04 μg), PA (0.02 μg), and NP (0.08 μg) from each virus [H3N2 Victoria, H5N1 (PB2-627K) 50-92, pH1N1 England 195, or IBV Florida 06] were transfected into 200,000 eHAP1 or A549 cells using Lipofectamine 3000 (Thermo Fisher) at ratios of 2 μl P3000 reagent per μg plasmid DNA and 3 μl Lipofectamine 3000 reagent per μg plasmid DNA. As reporter constructs, we transfected 0.04 μg PolI-luc, which encodes a minigenome containing a firefly reporter flanked by either influenza A or B promoter sequences, or, in Fig. 2e, PolI-BFP. pCAGGS-*Renilla* luciferase (0.04 μg) was transfected as a transfection and toxicity control. For exogenous expression, 0.1 μg pCAGGS plasmid encoding either the relevant FLAG-tagged *Anp32* gene or Empty pCAGGS was coexpressed with the RNP components. The ratio of transfected plasmids was constant at all times, namely 2:2:1:4:2:2:5 PB1:PB2:PA:NP:PolI reporter:*Renilla*:ANP32/Empty (if present). At least 20 h posttransfection, cells were lysed in 100 μl passive lysis buffer (Promega), and the dual-luciferase reporter assay kit (Promega) was used to measure bioluminescence on a FLUOstar Omega plate reader (BMG Labtech). In the case of a PolI-BFP reporter, please refer to the “Fluorescence microscopy” paragraph below. All minigenome assays were repeated in triplicate at least twice.

### Fluorescence microscopy.

At least 200,000 cells were cultured on glass coverslips in 24-well plates and transfected or infected as described. Cells transfected with plasmids encoding fluorescent proteins (BFP, GFP, or mCherry) were fixed in 4% paraformaldehyde and then visualized. Cells transfected with plasmids encoding (FLAG-tagged) nonfluorescent proteins were fixed and permeabilized in 0.2% Triton X-100. Primary antibodies used were rabbit anti-FLAG F7425 (Sigma) or mouse anti-IAV NP (Abcam 128193). Secondary antibodies were goat α-rabbit Alexa Fluor-594 (catalog number ab150080, Invitrogen) and goat α-mouse Alexa Fluor-568 (catalog number A11031; Invitrogen). Coverslips were mounted on glass slides using Vectashield mounting medium (H-1000-10; Vector Laboratories). Cells were imaged with a Zeiss Cell Observer widefield microscope with ZEN Blue software, using a Plan-Apochromat ×100 1.40-numerical aperture oil objective (Zeiss), an Orca-Flash 4.0 complementary metal-oxide semiconductor (CMOS) camera (frame, 2,048 × 2,048 pixels; Hamamatsu), giving a pixel size of 65 nm, and a Colibri 7 light source (Zeiss). Channels acquired and filters for excitation and emission were 4′,6-diamidino-2-phenylindole (DAPI) (excitation [ex], 365/12 nm, emission [em] 447/60 nm), GFP (ex 470/40 nm, em 525/50 nm), and TexasRed (ex 562/40 nm, em 624/40 nm). All images were analyzed and prepared with Fiji software (43). For images in Fig. 2e and Fig. S5b, the detection limit was adjusted individually for each channel (taking care to remain well above control background level), while in Fig. 3e, where we are comparing relative levels of NP expression, the lower detection limit in the TexasRed channel was set equal to that of the DAPI channel.

### Influenza virus infection.

Cells were infected with virus diluted in serum-free IMDM or DMEM at 37°C (MOI as indicated in the text or relevant figure legends) and replaced with serum-free cell culture medium supplemented with 1 μg · ml^−1^ l-1-tosylamide-2-phenylethyl chloromethyl ketone (TPCK) trypsin (Worthington-Biochemical) after 1 to 2 h. Cell supernatants were harvested at indicated time points postinfection. Infectious titers were determined by plaque assay on MDCK cells. All virus infection assays were performed in triplicate at least twice; Fig. 3a to d and Fig. S4 show one representative triplicate assay.

### Safety/biosecurity.

All work with infectious agents was conducted in biosafety level 2 facilities, approved by the Health and Safety Executive of the United Kingdom and in accordance with local rules, at Imperial College London, United Kingdom.

### Viral RNA quantitation.

Total RNA from 200,000 to 250,000 PR8-infected eHAP1 cells was extracted using the RNeasy minikit (Qiagen), with 30 min of on-column DNase I treatment (Qiagen). RNA concentrations were established by spectrophotometry (NanoDrop; Thermo Scientific), and equal amounts (500 ng) were subjected to cDNA synthesis using RevertAid reverse transcriptase (Thermo Scientific). PR8 segment 6 (neuraminidase [NA]) RNA species (vRNA, cRNA, and mRNA) were isolated using 5′-tagged primers (44) GGCCGTCATGGTGGCGAATGAAACCATAAAAAGTTGGAGGAAG, GCTAGCTTCAGCTAGGCATCAGTAGAAACAAGGAGTTTTTTGAAC, and CCAGATCGTTCGAGTCGTTTTTTTTTTTTTTTTTGAACAGACTAC, respectively (tags underlined). Unique fragments of the NA gene were then amplified by real-time quantitative PCR using Fast SYBR green master mix (Thermo Scientific), using the following primers: GGCCGTCATGGTGGCGAAT and CCTTCCCCTTTTCGATCTTG (vRNA,148 bp), CTTTTTGTGGCGTGAATAGTG and GCTAGCTTCAGCTAGGCATC (cRNA, 108 bp), or CTTTTTGTGGCGTGAATAGTG and CCAGATCGTTCGAGTCGT (mRNA, 87 bp) Quantitative PCR analysis was carried out on a Viia 7 real-time PCR system (Thermo Fisher). Gene expression was calculated by normalizing target gene expression to threshold cycle (*C_T_*) values obtained in the mock-infected condition.

### Bioinformatics.

The alignment in Fig. 4b was made in Clustal Omega, using primary sequences from UniProt (human ANP32A, accession number P39687; human ANP32B, Q92688; mouse Anp32A, O35381; and mouse Anp32B, Q9EST5).

### Structural modeling.

To illustrate a chimeric construct with the LRR 5 from murine Anp32B in murine Anp32A we created a homology model of MusB obtained using iTASSER structural prediction software (based primarily on huANP32B [GenBank accession number 2RR6A], huANP32A [accession number 2JQDA], and 2JEOA). The three-dimensional structural model was visualized and created in UCSF Chimera; the LRR is shown in dark gray and the structurally unresolved LCAR in semitransparent gray. Amino acid residues 128 to 153 are highlighted in blue and residue 130 is in red stick format.

### Data availability.

NGS reads were deposited in the European Nucleotide Archive and NCBI BioProject database under project accession number PRJEB31093.
